# Green Synthesis of Gold Nanoparticles Using Peach Extract Incorporated in Graphene for the Electrochemical Determination of Antioxidant Butylated Hydroxyanisole in Food Matrices

**DOI:** 10.3390/bios13121037

**Published:** 2023-12-18

**Authors:** Emanuela Grechi Döll, Edson Roberto Santana, João Paulo Winiarski, Luan Gabriel Baumgarten, Iolanda Cruz Vieira

**Affiliations:** Laboratory of Biosensors, Department of Chemistry, Federal University of Santa Catarina, Florianópolis 88040 900, Santa Catarina, Brazil; emanuelagrechi@gmail.com (E.G.D.); joao.winiarski@ufsc.br (J.P.W.); baumgarten.luan@posgrad.ufsc.br (L.G.B.); iolanda.vieira@ufsc.br (I.C.V.)

**Keywords:** green synthesis, electrochemical sensor, food additive, BHA, antioxidant

## Abstract

Butylated hydroxyanisole (BHA) is a synthetic phenolic antioxidant widely used in various food matrices to prevent oxidative rancidity. However, its presence has been associated with liver damage and carcinogenesis in animals. Thus, an electrochemical sensor was built using a composite of gold nanoparticles synthesized in peach extract (*Prunus persica* (L.) Batsch) and graphene. Peach extract served as a reducing and stabilizing agent for gold nanoparticles, as a dispersing agent for graphene, and as a film former to immobilize the composite on the surface of a glassy carbon electrode. The gold nanoparticles were characterized using spectroscopic and microscopic techniques, and the electrodes were electrochemically characterized using electrochemical impedance spectroscopy and cyclic voltammetry. The sensor provided higher current responses and lower charge transfer resistances compared to the unmodified glassy carbon electrode. Under the established optimized working conditions (0.1 mol L^−1^ Britton–Robinson buffer, pH 4.0, and differential pulse voltammetry), the calibration curve exhibited a linear range from 0.2 to 9.8 µmol L^–1^, with a detection limit of 70 nmol L^−1^. The proposed sensor represented a sensitive and practical analytical tool for the accurate determination of BHA in mayonnaise samples.

## 1. Introduction

Antioxidants have played a crucial role in the food industry for over 50 years, acting as additives to prevent or delay lipid oxidation. Lipid oxidation is responsible for the development of unpleasant flavors and odors in food, making them unfit for consumption [1]. To extend the shelf life of food products, manufacturers often turn to synthetic phenolic antioxidants (SPAs), such as butylated hydroxyanisole (BHA), one of the main additives used in the food industry [2,3]. Commercially available BHA usually consists of two isomers: 10% 2-tert-butyl-4-hydroxyanisole (2-BHA) and 90% 3-tert-butyl-4-hydroxyanisole (3-BHA). This antioxidant can be added alone or in combination in oils and fats, seasonings, animal feed, chewing gum, and other food supplements [3].

Although SPAs are generally recognized as safe at authorized levels, their use is not without problems, as their toxicological effects have been observed in in vitro and in vivo studies. A study conducted by Yang et al. (2018) [4], for example, revealed that BHA can trigger endocrine disorders at low concentrations (1.0 μmol L^−1^) and is also associated with liver damage and carcinogenesis in laboratory animals. Furthermore, other studies have identified the presence of synthetic phenolic antioxidants in human samples, such as adipose tissues, serum, urine, breast milk, and nails [5].

For these reasons, the use of synthetic antioxidants in food is strictly regulated by specific legislation in different countries. To ensure compliance with legal requirements and quality control standards in the food industry, precise determination of SPAs in food is essential [6]. Currently, analysis of antioxidant compounds is mainly conducted by methods such as high-performance liquid chromatography coupled with mass spectrometry [7], high-performance liquid chromatography with ultraviolet detection [8], and capillary electrophoresis with amperometric detection [9]. However, these methods require sample pretreatment (such as preconcentration, extraction, and dilution). Moreover, chromatographic separation coupled with different methods is costly and requires long run times. Spectrophotometric methods, due to their simplicity, used to be a popular analytical technique. However, these methods are subject to severe matrix interference.

To ensure compliance of food products with acceptable quality standards for consumption, it is essential to have an analytical method that is simple, reproducible, and rapid [10,11,12]. In this context, electrochemical sensors emerge as a promising analytical strategy due to their ease of use, affordability, and ability to analyze samples directly without the need for expensive pretreatments. To further enhance the performance of these sensors, new materials have been employed in their construction, such as polymers, surfactants, metallic nanoparticles, and nanostructured carbon materials [13]. Several electrochemical sensors based on nanomaterials have been developed to determine BHA in different matrices [14,15,16,17].

Gold nanoparticles (AuNPs) are attractive for the development of electrochemical sensors due to their unique properties. They exhibit a high surface area-to-volume ratio, which increases sensitivity in detecting target molecules. Additionally, AuNPs are known for their stability and excellent biocompatibility, making them a promising choice for creating new chemical and biological devices [18].

The most common approach for producing AuNPs is chemical synthesis. However, this method involves the use of costly and potentially toxic chemicals, limiting its application in areas such as biology and medicine, as well as having a negative impact on the environment [19]. For this reason, efforts have been made to develop more sustainable processes. In this regard, the synthesis of metal nanoparticles using plant extracts has attracted attention due to its simplicity, economic feasibility, and environmental sustainability [20,21]. Plant extracts contain various substances such as enzymes, proteins, amino acids, polysaccharides, phenols, tannins, and other metabolites that act as reducing agents in nanoparticle formation. Moreover, the biocompounds present in plants can form a stabilizing layer around the nanoparticles, preventing their agglomeration and ensuring their stability over time [22]. The plant-mediated synthesis of nanomaterials stands out as the most efficient method, primarily attributed to the high yield achieved. This elevated yield is a consequence of the remarkable stability exhibited by the synthesized nanomaterials across various plant species. The diverse array of metabolites and biochemical compounds within plants plays a crucial role in stabilizing these nanomaterials. Consequently, the plant-mediated synthesis of nanomaterials emerges as both a cost-effective and environmentally friendly approach [23].

Among the various possible bioreducers, peach extract is a possibility to be explored. Peach (*Prunus persica* (L.) Batsch) is part of the Rosaceae family and is one of the world’s most economically important fruit crops, with annual global production of over 20 million tons [24,25]. In this regard, Kumar et al. [26] reported the green synthesis of silver nanoparticles using *Prunus persica* leaf extract, while Skiba and Vorobyova [27] used peach pomace for the same purpose. However, peach pulp is still not widely explored. Peach pulp is rich in water, sugar, proteins, vitamins, and minerals, as well as phenolic compounds such as chlorogenic acid, caffeic acid, catechin, epicatechin, rutin, and cyanidin-3-glucoside, which can act as reducing and stabilizing agents in green synthesis [28,29,30].

In addition to metallic nanoparticles, graphene is a carbon allotrope that arouses great interest for electrochemical applications due to its planar structure and high electrical conductivity, offering advantages over other materials used in sensor construction [31]. Functionalization of graphene with nanoparticles has been widely explored to enhance its properties, including sensitivity, detection limit, and reproducibility, resulting in a more robust analytical response of the sensors [32]. In that regard, Wang et al. [33] used a nanocomposite with Au NPs and graphene oxide stabilized in polyvinylpyrrolidone to develop a sensor for BHA. Au NPs were synthesized using ascorbic acid as a reducing agent at 90 °C.

In this study, the use of peach pulp extract as a reducing and stabilizing agent in the synthesis of AuNPs was proposed, along with the incorporation of graphene to improve the performance of the electrochemical sensor. The nanocomposite was prepared at room temperature (25 °C), with peach extract being the agent responsible for the entire process. Furthermore, the peach extract allowed the formation of a stable film on the surface of a glassy carbon electrode (GCE), used as a substrate for the sensor. Thus, the aim of this study was to develop an efficient and sustainable analytical method for the determination of BHA in food matrices.

## 2. Materials and Methods

### 2.1. Reagents and Solutions

All chemicals were of analytical grade and used without further purification. BHA, potassium ferricyanide, potassium ferrocyanide, and chloroauric acid (HAuCl_4_) were obtained from Merck (Darmstadt, Germany). Graphene was purchased from 2DM, Singapore. The solutions were prepared using ultrapure water (18.2 MΩ cm, Milli-Q system, Millipore, Burlington, MA, USA). Solutions of BHA were prepared daily at a concentration of 1.0 mmol L^−1^. Britton-Robinson buffer (CH_3_COOH/H_3_PO_4_/H_3_BO_3_) was used in pH studies.

### 2.2. Biosynthesis and Characterization of Peach Extract-Stabilized Gold Nanoparticles (AuNP-Peach-ext)

To prepare the peach extract, the fruit (*Prunus persica* (L.) Batsch) was initially peeled and 5.0 g, approximately, was weighed and macerated with 20.0 mL of ultrapure water. Subsequently, the obtained extract was centrifuged at 10,000 rpm for 3 min at 25 °C, resulting in a clear dispersion [34]. The extract was used as both a reducing and stabilizing agent in the biosynthesis of AuNPs.

After this procedure, 1.0 mL peach extract dispersion (Peach-ext) and 2.25 mL of ultrapure water were mixed in a beaker with stirring (500 rpm) at room temperature (25 °C). Then, 0.25 mL of the 6.0 mmol L^−1^ HAuCl_4_ was quickly added to the dispersion, which was kept under agitation for about 3 min, resulting in a reddish coloration, confirming the formation of gold nanoparticles (AuNP-Peach-ext).

The characterization of Peach-ext and AuNP-Peach-ext was performed using UV-Vis spectroscopy on a Cary 60 UV-Vis spectrometer (Agilent Technologies, Santa Clara, CA, USA). Similarly, dispersions also were characterized by attenuated total reflectance Fourier transform infrared (ATR-FTIR) spectroscopy using a Spectrum-100 spectrometer (PerkinElmer, Waltham, MA, USA). For ATR-FTIR analysis, solids resulting from centrifugation (10,000 rpm, 2 °C, 40 min) and drying (45 °C, 24 h) of the dispersions were analyzed.

### 2.3. Preparation and Characterization of Gr-AuNP-Peach-ext

The dispersion of graphene-gold nanoparticles immobilized in peach pulp extract (Gr-AuNP-Peach-ext) was prepared by mixing 100 μL of AuNP-Peach-ext and 0.5 mg of graphene (Gr). An ultrasonic bath was utilized for the homogenization of the dispersion (60 s). A JEM-1011 microscope (JEOL, Akishima, Tokyo, Japan) at an accelerating voltage of 100 keV was utilized for the microscopic analysis. For this, the AuNP-Peach-ext was deposited on carbon-coated copper grids (300 mesh) and then dried in the air.

### 2.4. Preparation of Sensors, Electrochemical Measurements and Data Analysis

To prepare the sensor, a GCE (0.03 cm^2^) was first mechanically polished. The surface was modified with 2.0 μL of Gr-AuNP-Peach-ext dispersion using a micropipette. After drying the film in a vacuum desiccator (15 min), the sensor was used as the working electrode.

Electrochemical analyses were performed using a PGSTAT101 potentiostat (Metrohm Autolab B.V., Utrecht, The Netherlands). The electrochemical cell consisted of a working electrode (Gr-AuNP-Peach-ext/GCE or AuNP-Peach-ext/GCE or GCE bare), a reference electrode (Ag/AgCl with 3.0 mol L^−1^ KCl), and a counter electrode (platinum plate). The measurements using voltammetric techniques were performed at room temperature (25 °C) in an electrochemical cell containing 15.0 mL of supporting electrolyte (0.1 mol L^−1^ B-R buffer; pH 4.0) and a specific volume of the analyte, BHA, according to each analysis. The voltammograms were recorded after magnetic stirring for sixty seconds, the time required for solution homogenization in the cell. Cyclic voltammetry was performed in a potential range of −0.3 to +0.8 V, with a scan rate of 50 mV s^−1^. Differential pulse voltammetry (DPV) measurements were performed in a potential range of +0.3 to +0.7 V, scan rate of 60.0 mV s^−1^, pulse amplitude of 100 mV, and pulse duration of 5.0 ms.

A PGSTAT128N potentiostat (Metrohm Autolab B.V., Utrecht, The Netherlands) was used to characterize the different electrodes by electrochemical impedance spectroscopy (EIS) in a frequency range of 0.1–50,000 Hz at the open circuit potential. Spectra were recorded using an equimolar solution of 5.0 mmol L^−1^ of K_3_[Fe(CN)_6_]/K_4_[Fe(CN)_6_] in 0.1 mol L^−1^ KCl.

### 2.5. Sample Preparation

To prepare the sample, 1.0 g of store-bought mayonnaise was mixed with 2.0 mL of ethanol in a test tube. The mixture was then sonicated for 30 min in an ultrasonic bath. Subsequently, the homogenized mixture was centrifuged at 3000 rpm for 5 min. This extraction procedure was repeated twice. The extracts were collected and diluted to 5.0 mL with ethanol [35]. An aliquot of 1.0 mL of the sample solution was introduced into the electrochemical cell containing 9.0 of 0.1 mol L^−1^ B-R buffer (pH 4.0) and was analyzed using the optimized DPV conditions.

## 3. Results and Discussion

### 3.1. Synthesis and Characterization of AuNP-Peach-ext Dispersion

Initially, the peach extract showed a clear dispersion. After the addition of HAuCl_4_ solution, the dispersion turned a reddish coloration within three minutes, indicating the occurrence of the reducing reaction of the gold metallic ion (Au^3+^) by the bioactive compounds present in the extract forming the gold nanoparticles. To this end, the extract acted as both as reducing and stabilizing agent in the biosynthesis of AuNPs. Previous studies have shown that peaches contain a variety of phenolic compounds, such as chlorogenic acid, catechins, epicatechins, and anthocyanins, along with polyphenol oxidase enzyme, which are likely involved in the reduction in the metallic substrates to form metallic nanoparticles [26,36].

**Figure 1:**
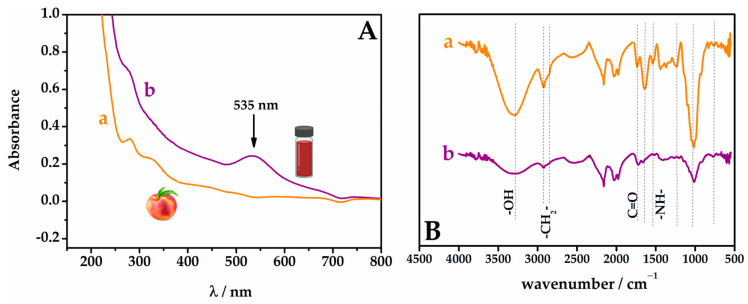
(**A**) UV-Vis absorbance spectra and (**B**) ATR-FTIR spectra for (a) pure *Prunus persica* (L.) Batsch pulp extract and (b) AuNPs synthesized from *Prunus persica* (L.) Batsch pulp extract.

Gold nanoparticles are known to exhibit colors ranging from red to violet, depending on their size. The observed color change confirmed the formation of the AuNPs was further supported by the UV-Vis analysis (Figure 1A). The spectrum revealed that the synthesized gold nanoparticles absorb light in the wavelength region of 535 nm, which is characteristic of the surface plasmon resonance bands of AuNPs with an average size of 2 to 40 nm [37]. Indeed, the absorption bands of peach extract changed from the spectrum of peach extract (Figure 1A—curve a), which could be related to the change in transitions of *n*-π and π-π* of phenolic compounds [38] for the bands of respective quinones, observed after bioreduction of Au^3+^ (Figure 1A—curve b).

The ATR-FTIR spectra of pulp peach extract and pulp peach extract with gold nanoparticles are presented in Figure 1B. In the spectrum corresponding to the peach fruit (Figure 1B—spectrum a), a broad band at approximately 3288 cm^−1^ was observed, assigned to the stretching vibration of hydroxyl functional groups. The bands at 2917 and 2849 cm^−1^ were attributed to the asymmetric and symmetric stretching vibrations of methylene groups, the most recurrent structural unit in the components of peach extract, exhibiting distinct features of long-chain aliphatic compounds. The robust absorption band at 1731 cm^−1^ is assigned to the C=O stretching vibration of the carbonyl group. Absorptions recorded from 1650 to 1500 cm^−1^ correspond to the designated aromatic domain, predominantly composed of phenolic compounds. The absorption band at 1535 cm^−1^ is assigned to the free -NH- groups present in polysaccharides and proteins, while the band at 1015 cm^−1^ is indicative of glycosidic bonds typical of polysaccharides [39,40]. The spectrum of AuNP-Peach-ext (Figure 1B—spectrum b) exhibits similar bands to those of peach extract. However, a significant difference is noted for two bands around 3292 cm^−1^, where the band in AuNP-Peach-ext is smaller than that in the pure extract. This suggested that the -OH groups present in the peach extract were the primary compounds involved in the reduction in Au ions. The band at 1635 cm^−1^ indicated the contributory roles of C=O bonds in the reduction and stabilization of Au^3+^ ions to Au^0^. Consequently, phenolic, alcoholic, and carboxylic compounds were considered accountable for the reduction and stabilization of AuNP-Peach-ext. Furthermore, a decreased peak intensity at 763 cm^−1^ suggested the binding between the C-H group of phenolic acids and AuNPs. The reduction in this band’s intensity after the formation of AuNPs indicated that these biomolecules in the extract were also utilized for capping AuNPs, thereby improving their stability [41,42].

**Figure 2:**
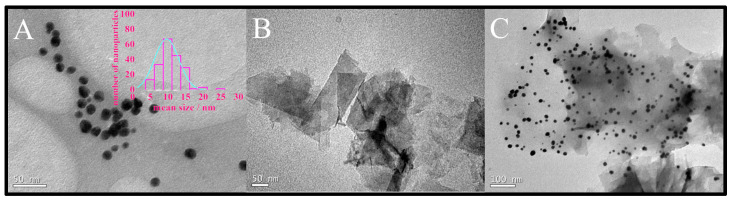
Transmission electron microscopy of (**A**) AuNP-Peach-ext; (**B**) Gr-Peach-ext; and (**C**) Gr-AuNP-Peach-ext. Inset: histogram of mean size of nanoparticles.

To understand the morphologies and size distribution of AuNP-Peach-ext and Gr-AuNP-Peach-ext, the samples were imaged using TEM (Figure 2). TEM images of the samples showed that AuNP-Peach-ext was roughly spherical in shape with an average diameter of around 11.5 ± 1.5 nm (Figure 2A). Furthermore, to assess the dispersion capability of peach extract, graphene was introduced (Figure 2B). The well-dispersed nature of graphene sheets in the peach extract is evident, likely attributed to potential π–π interactions between the extract and the carbonaceous material. The AuNP-Peach-ext dispersion was well distributed in graphene sheets (Figure 2C) owing to interactions of peach extract with graphene sheets, showing that gold nanoparticles are supported in the graphene due mainly to interactions of the peach extract with the carbon structure of graphene. The size distribution of the Gr-AuNP-Peach-ext was also counted and the average diameter of AuNP had not changed, making it clear that graphene was a great support for gold nanoparticles stabilized and dispersed by the peach extract.

### 3.2. Electrochemical Characterization of Modified Electrodes

All surface modification steps studied were monitored using the electrochemical techniques of CV and EIS on a newly developed electrode, with the aim of evaluating the promising potential of this new device for electroanalytical applications. The system used was potassium ferricyanide/ferrocyanide ([Fe(CN)_6_]^3−^/[Fe(CN)_6_]^4−^), which exhibits a reversible electron-transfer process controlled by diffusion and is already known in the literature [43].

**Figure 3:**
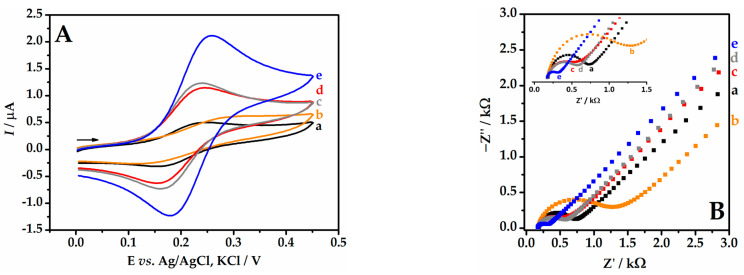
(**A**) Cyclic voltammograms for 5.0 mmol L^−1^ K_4_[Fe(CN)_6_] in 0.1 mol L^−1^ KCl (ν = 50 mV s^−1^) on different electrodes. (**B**) Nyquist plots using an equimolar mixture of 5.0 µmol L^−1^ [Fe(CN)_6_]^3−^/[Fe(CN)_6_]^4−^ in 0.1 mol L^−1^ KCl with different electrodes: (a) GCE; (b) Peach-ext/GCE; (c) AuNP-Peach-ext/GCE; (d) Gr-Peach-ext/GCE; and (e) Gr-AuNP-Peach-ext/GCE.

Figure 3A presents the cyclic voltammograms for the different electrodes: GCE, ext/GCE, AuNP-ext/GCE, Gr-ext/GCE, and Gr-AuNP-ext/GCE. The unmodified GCE (curve a) showed the lower anodic (*I*_pa_) and cathodic (*I*_pc_) peaks’ current intensities. With the modification of the peach extract film (curve b), the data worsened, since the extract is not a conductive substance. When the AuNP-Peach-ext/GCE (curve c) and Gr-Peach-ext/GCE (curve d) were used, an increase in both currents compared to the bare GCE was observed. A further enhanced response was noted for the sensor containing graphene and AuNPs (curve e), which can be attributed to the high conductivity of both modifiers and the synergistic effect when combined.

Similar behaviors related to the electrode modification steps were observed in the EIS study. From this study, it was possible to extract the charge transfer resistance (R_ct_), which corresponds to the diameter of the semicircle in the Nyquist plot (Figure 3B). After coating the electrode with the peach extract (curve b), an increase in the R_ct_ was observed, with the highest value recorded in this study (R_ct_ = 1330 Ω), even higher than the unmodified GCE (curve a, R_ct_ = 669 Ω). This resistive behavior of Peach-ext/GCE can be explained by the non-conducting nature of the plant extract. When the electrode was prepared with AuNPs (curve c), there was a decrease in the charge transfer resistance (R_ct_ = 586 Ω), which also occurred when the electrode was modified with graphene (curve d, R_ct_ = 515 Ω). These decreases were a result of the conductive interface of the AuNPs and graphene. Lastly, the most significant contribution to the improvement in electron transfer in the system was observed in the presence of graphene with gold nanoparticles stabilized in peach extract (curve e). A low charge transfer resistance was observed for this electrode (R_ct_ = 219 Ω), demonstrating a great advantage in the proposed modification, as the reduction in resistance facilitates electron transfer on the electrode surface and, consequently, the oxidation reaction of the analyte.

It is important to note that the trends observed in CV were reinforced by EIS data. The electrode modified with graphene and AuNPs exhibits lower charge transfer resistances and higher current intensities, demonstrating better electroactivity compared to the bare GCE. This characteristic makes this device promising for electroanalytical applications.

### 3.3. Electrochemical Investigation of BHA

**Figure 4:**
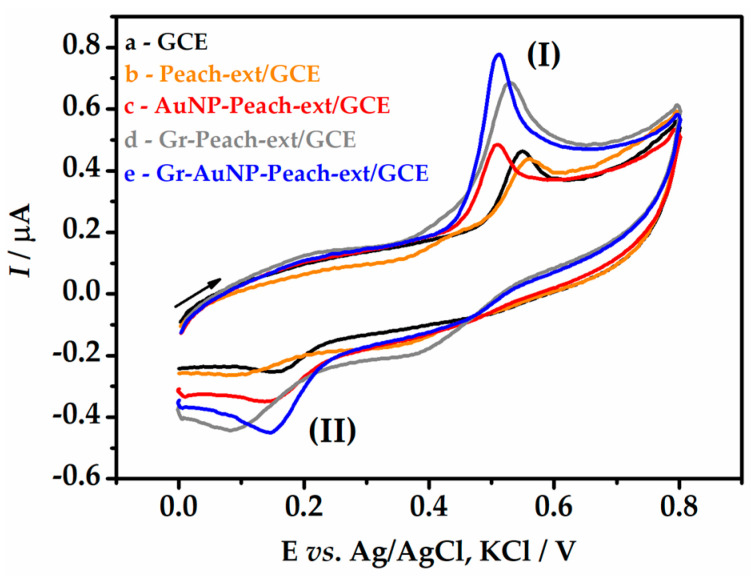
Cyclic voltammograms for 1.0 µmol L^−1^ BHA in 0.1 mol L^−1^ B-R buffer (pH 3.0) using different electrode configurations. ν = 50 mV s^−1^.

The electrochemical behavior of BHA at different electrode modification steps was investigated using CV. Cyclic voltammograms for BHA on all tested electrodes exhibit well-separated oxidation (I) and reduction (II) peaks (Figure 4). The separation between these peaks (ΔE_p_ ~ 0.38 V) was greater than the value of (0.0592/z) V, where z is the number of transferred electrons, which in the case of BHA is z = 2 [44]. Therefore, the electrochemical behavior of BHA on the tested electrodes was considered irreversible.

Regarding current intensities, low values were obtained with the unmodified GCE (curve a), with *I*_pa_ = 0.18 µA and *I*_pc_ = −0.06 µA (ΔE_p_ = 0.38 V). With the electrode modified with peach extract (curve b), there was a slight decrease in current intensities, since the fruit extract is not a conductive substance, which hindered the charge transfer reaction (ΔE_p_ = 0.45 V). With the incorporation of AuNPs into the electrode (curve c), the current intensities increased by about 1.5 times (ΔE_p_ = 0.31 V) due to the electrocatalytic conductive properties of AuNPs. The electrode containing only graphene in the peach extract (curve d) provided an enhancement of two-fold the current responses compared to AuNP-Peach-ext/GCE; however, it hindered charge transfer (ΔE_p_ = 0.42 V).

Finally, the Gr-AuNP-Peach-ext/GCE (curve e) exhibited current intensities approximately four times higher than the unmodified GCE, resulting from the conductive properties of graphene and AuNPs, with ΔE_p_ = 0.32 V. Therefore, the Gr-AuNP-Peach-ext/GCE electrode and the oxidation peak (I) were selected for subsequent studies with the aim of developing a sensitive electroanalytical method for BHA monitoring.

### 3.4. Study of pH Value on the BHA Oxidation

**Figure 5:**
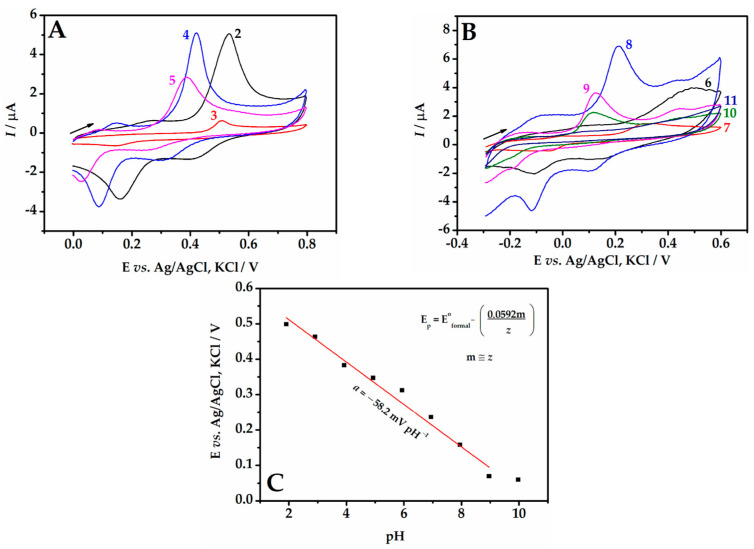
Cyclic voltammograms for 1.0 µmol L^−1^ BHA using the Gr-AuNP-Peach-ext/GCE in B-R buffer (0.1 mol L^−1^) at different pH values: (**A**) pH 2.0 to 5.0 and (**B**) pH 6.0 to 11.0. (**C**) Relationship between E_p_ and pH. ν = 50 mV s^−1^.

The investigation of the pH of the supporting electrolyte is extremely important for the sensitivity of the sensors, as the acidity or alkalinity of the medium can affect how the electroactive species is present in the solution (protonated or deprotonated), altering the way the analyte will react on the electrode surface. These variations can result in changes in the current and potential values obtained in the voltammetric measurement, which can negatively impact the method’s sensitivity. In order to investigate the effect of pH on the electrochemical response of BHA in B-R buffer, CV experiments were performed at pH ranging from 2.0 to 11.0 (Figure 5A,B). It can be observed that the potential shifts to more negative values as the solution pH values increase, favoring the redox reaction. The highest peak current intensity was obtained at pH 4.0, and this value was adopted for subsequent analyses aiming for improved sensitivity in the determination of the analyte.

Figure 5C shows the relationship between the peak potential (E_p_) and pH. The results indicated a linear relationship with a slope of −58.3 mV pH^−1^. This suggests that the oxidation/reduction process of BHA involved the same number of protons and electrons, close to the theoretical Nernst value (−59.2 mV pH^−1^) described in previous studies [35,45]. Furthermore, the intersection of the line occurred around pH 9.0, which was consistent with the theoretical pKa value of 8.90 for the deprotonation of BHA [6].

### 3.5. Behavior of BHA at Different Scan Rate

**Figure 6:**
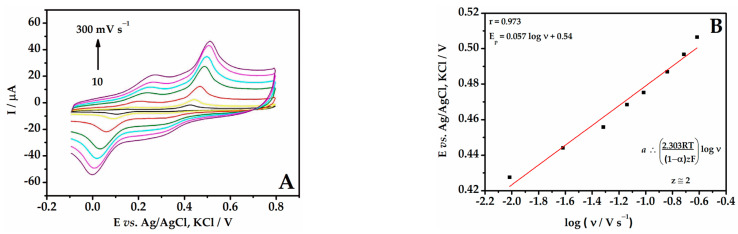
(**A**) Cyclic voltammograms for 1.0 µmol L^−1^ BHA on the Gr-AuNP-Peach-ext/GCE in 0.1 mol L^−1^ B-R buffer (pH 4.0) at different scan rates: 10, 25, 50, 75, 100, 150, 200, 250, and 300 mV s^−1^. (**B**) Relationship between E_p_ vs. log *v*.

Cyclic voltammograms were recorded at different scan rates (10–300 mV s^−1^) in order to study the BHA processes at the electrode surface (Figure 6A). The results showed that increasing the scan rate resulted in an increase in the current of the anodic and cathodic peaks. Additionally, the peak potentials were shifted and the distance between the peaks increased, indicating the irreversibility of the system.

**Figure 7:**
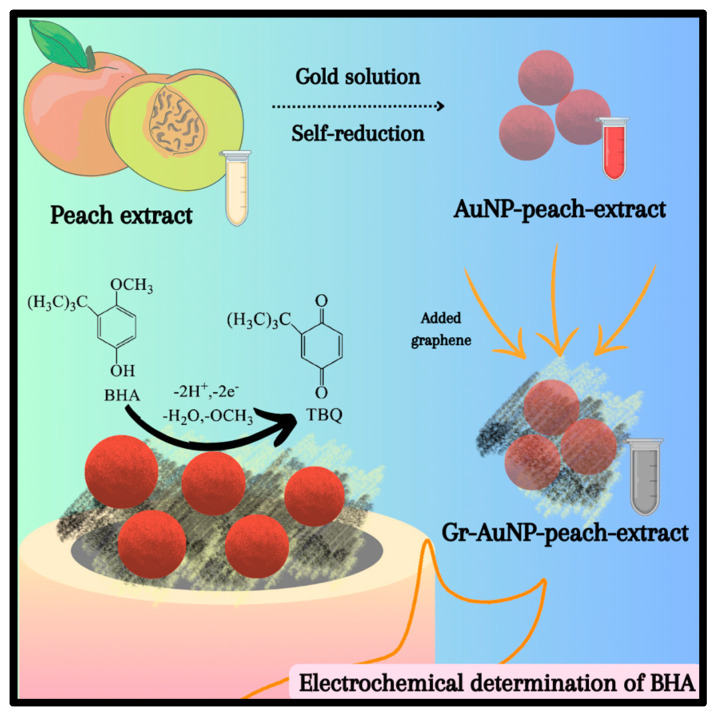
Schematic BHA oxidation reaction onto Gr-AuNP-Peach-ext/GCE.

In Figure 6B, it can be observed that the relationship between the peak potential (E_p_) and the logarithm of the scan rate (log *v*) was linear (E_p_ = 0.057 log *v* + 0.54). Using Laviron’s equation and considering an irreversible process, the number of apparent electrons involved (z_app_) in the oxidation of BHA was estimated close to 2. The results obtained in Section 3.4 showed that the same number of moles of protons and electrons were involved in the oxidation reaction of BHA. Therefore, it can be stated that the reactions involved two moles of protons and two moles of electrons per mole of BHA, as observed in the reaction shown in Figure 7. This proposed reaction is consistent with the literature [46].

### 3.6. Differential Pulse Optimization Parameters

In order to develop a sensitive method for the quantification of BHA, three different electroanalytical techniques and the magnitude of the analytical signal provided by them were investigated. The performance of linear sweep voltammetry (LSV), differential pulse voltammetry (DPV), and square wave voltammetry (SWV) was evaluated. The differential pulse technique showed the highest current response for the analyte and was chosen for further studies.

To optimize the differential pulse voltammetry technique, several instrumental parameters such as potential scan rate (*v*), potential pulse amplitude (E_pulse_), and pulse duration time (t_pulse_) were evaluated to obtain the maximum current values for the proposed sensor. The scan rate was varied from 10.0 to 100 mV s^−1^, while keeping E_pulse_ = 50.0 mV and t_pulse_ = 10.0 ms. The highest current intensity was obtained at a scan rate of 60 mV s^−1^, which allowed sensitive monitoring of the reaction kinetics. The pulse duration time (t_pulse_) was evaluated in the range of 1.0 to 30.0 ms, while keeping E_pulse_ = 50.0 mV and *v* = 60 mV s^−1^. A time of 5.0 ms was selected, as it exhibited the highest response current for BHA. The pulse amplitude was investigated in the range of 10 to 120 mV, while keeping t_pulse_ = 5.0 ms and *v* = 60 mV s^−1^. An amplitude of 100 mV was chosen as there was a linear growth of peak current intensity up to this point, remaining constant at higher values.

### 3.7. Calibration Plot

**Figure 8:**
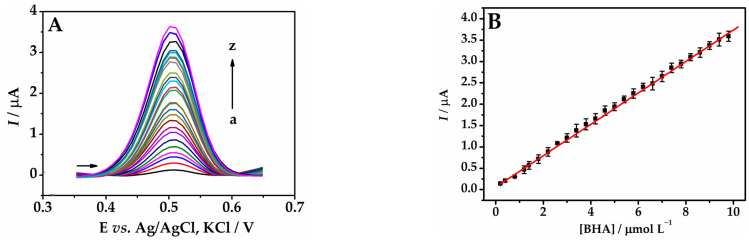
(**A**) Differential pulse voltammograms for BHA on the Gr-AuNP-Peach-ext/GCE at different concentrations: (a) 0.2, (b) 0.4, (c) 0.8, (d) 1.2, (e) 1.4, (f) 1.8, (g) 2.2, (h) 2.6, (i) 3.0, (j) 3.4, (k) 3.8, (l) 4.2, (m) 4.6, (n) 5.0, (o) 5.4, (p) 5.8, (q) 6.2, (r) 6.6, (s) 7.0, (t) 7.4, (u) 7.8, (v) 8.2, (w) 8.6, (x) 9.0, (y) 9.4, and (z) 9.8 µmol L^−1^ in 0.1 mol L^−1^ B-R buffer (pH 4.0). (**B**) Calibration plot of BHA (*n* = 3). DPV parameters: scan rate of 60.0 mV s^−1^, pulse amplitude of 100 mV, and pulse duration of 5.0 ms.

The calibration plot for BHA was constructed, registering a well-defined peak at +0.50 V vs. Ag/AgCl, KCl corresponding to the oxidation of BHA (Figure 8A). The linear regression (Figure 8B) can be expressed as follows: *I* = 0.36 (±0.002) [BHA] + 0.098 (±0.008) (r = 0.998) for the concentration range of 0.2 to 9.8 µmol L^−1^. The limits of detection [LOD = 3 × (SD/*s*)] and quantification [LOQ = 10 × (SD/*s*)] were 0.07 and 0.22 µmol L^−1^, respectively, being obtained through the parameters of the calibration plot: SD (standard deviation) of the intercept and *s* the slope of the calibration plot [47].

According to the results, the proposed sensor demonstrated advantageous aspects compared to other studies dedicated to BHA quantification. The detection limit obtained is one of the lowest compared to the selection of sensors listed in Table 1. However, the green chemistry surrounding the use of peach extract is the highlight of this sensor. Without the need to use toxic reagents, AuNPs were synthesized using peach extract as a reducing agent. Furthermore, the natural extract served as a dispersing agent for graphene, forming a conductive nanocomposite. And finally, the peach extract still formed a stable film on the surface of the GCE. Therefore, the sensor in question has great potential for application in the determination of BHA in food samples, providing sensitive data, and it can be obtained with natural and low-cost reagents.

### 3.8. Precision, Interferents, and Stability Data

The precision of the Gr-AuNP-Peach-ext/GCE response for BHA assays was evaluated by measuring the repeatability of the current response of 1.4 μmol L^−1^ BHA in 0.1 mol L^−1^ B-R buffer (pH 4.0). The values for the relative standard deviation (RSD) of the DPV current were measured on the same day with different films (intra-day repeatability) (*n* = 5) and was 4.17%. The RSD of five measurements on consecutive days (inter-day repeatability) was 7.6%. The results showed that the prepared sensor had an acceptable precision because the obtained values were lower than ^2^/_3_RSD_Horwitz_ (RSD_Horwitz_ = 2^(1 − 0.5log C)^, in which C is the analyte mass fraction g g^–1^) [47].

Some interferent compounds were tested in the BHA electrochemical response, such as sodium benzoate, starch, citric acid, and lactose, because these are commonly used in mayonnaise [49]. The interference study results showed that the Gr-AuNP-Peach-ext/GCE sensor maintained its effective activity for the oxidation of BHA in the presence of these interfering substances, demonstrating its good selectivity. Therefore, the developed electrochemical sensor can be used for the analysis of BHA in food matrices.

The stability of the Gr-AuNP-Peach-ext dispersion was investigated over a two-month period. The current responses provided by Gr-AuNP-Peach-ext with the same dispersion for the BHA decreased by about 8% after two months when compared with the first current response, indicating that the Gr-AuNP-Peach-ext dispersion remained stable for the electrochemical application during this time. These results are in accordance with the literature, which reports that AuNPs stabilized in plant extract remain stable for 2 to 3 months [21].

The stability of the Gr-AuNP-Peach-ext film on the GCE surface was also evaluated by performing successive measurements of 1.4 μmol L^−1^ BHA using the same film. The decrease in current response to the BHA reaction after six successive measurements was 5.1% in comparison with the first measurement. This phenomenon could be associated with leaching of the film to the supporting electrolyte or the blockage of the surface by BHA oxidation products. Therefore, the number of measurements carried out with the same modified electrode was limited to six. After this, the surface of the electrode was cleaned and a new film was prepared.

### 3.9. Determination of BHA in Mayonnaise Samples

The Gr-AuNP-Peach-ext/GCE was implemented in the quantification of BHA in three samples of mayonnaise (Table 2). The obtained recoveries (92% to 105%) demonstrated that the sensor generated reliable results, suggesting that the analytical method was accurate.

Mayonnaise is a low-pH oil in water emulsion with a high concentration of oil. Thus, mayonnaise is susceptible to deterioration due to auto-oxidation of the unsaturated fats in the oil. One of the solutions for slowing lipid oxidation is the addition of antioxidants to the composition of the food. [55]. Thus, adding BHA to mayonnaise to act as an antioxidant is quite common [55]. In Brazil, the use of these antioxidants is controlled by The National Health Surveillance Agency (ANVISA), which limits the amount to 200 mg kg^–1^ for BHA [56]. The values determined using the sensor were 10 times less than the legal limit. These values corroborated data from Medeiros, Rocha-Filho, and Fatibello-Filho [46], who determined BHA in mayonnaise samples from Brazilian companies using a boron-doped diamond electrode and found values in the range of 17.0 to 23.0 mg kg^–1^. Thus, we can conclude that Gr-AuNP-Peach-ext/GCE was successfully applied to determine BHA in food matrices.

## 4. Conclusions

The present study demonstrated a simple, fast, and cost-effective route for synthesizing gold nanoparticles using *Prunus persica* (L.) Batsch extract. The plant extract acted as a reducing agent for the synthesis of AuNPs, as a dispersing agent for Gr, and as a film-former for the preparation of the electrochemical sensor. The integration of graphene with gold nanoparticles improved the current response of the sensor for BHA determination. Cyclic voltammetry studies concluded that the oxidation process of BHA was irreversible, and involved the exchange of two moles of electrons and two moles of protons per mole of BHA. The sensor exhibited favorable analytical characteristics under the established optimal working conditions (0.1 mol L^−1^ B-R buffer, pH 4.0), including a good linear range (0.2 to 9.8 µmol L^−1^), a LOD of 70 nmol L^−1^, and applicability in BHA determination in food matrices.

## Figures and Tables

**Table 1:** Comparison of analytical performance of Gr-AuNP-Peach-ext/GCE with other electrodes in the determination of BHA.

Electrodes	Technique	pH	LOD/nmol L^−1^	Reference
SPE-MWCNT ^a^	LSV	B-R buffer (pH 2.0)	176	[48]
MCCE-Cu_3_(PO_3_)_2_-Poly ^b^	SWV	KNO_3_/10% ethanol (*v*/*v*) (pH 6.7)	72	[49]
AuNP/graphite	LSV	B-R buffer (pH 2.0)	550	[50]
Gr/Ch/GCE ^c^	DPV	Phosphate buffer (pH 3.0)	190	[51]
CoHCF-graphite ^d^	Amperometry	Phosphate buffer (pH 7.0)	190	[52]
Au–PVP–GO/GCE ^e^	LSV	B-R buffer (pH 4.0)	40	[33]
MIP/MoS_2_/AgNPs-CS/GCE ^f^	DPV	Phosphate buffer (pH 7.0)	7.9	[53]
GR–PB/GCE ^g^	Amperometry	Phosphate buffer (pH 6.0)	76	[54]
Gr-AuNP-Peach-ext/GCE	DPV	B-R buffer (pH 4.0)	70	This work

^a^ Screen-printed electrode modified with multi-walled carbon nanotubes. ^b^ Composite electrode modified with copper (II) phosphate immobilized in polyester. ^c^ Glassy carbon electrode modified with choline monolayer and graphene. ^d^ Graphite and paraffin composite electrode modified with cobalt hexacyanoferrate. ^e^ Glassy carbon electrode modified with gold nanoparticles stabilized in PVP and graphene oxide. ^f^ Molecularly imprinted glassy carbon electrode modified with flower- like molybdenum disulfide and silver nanoparticle-chitosan. ^g^ Glassy carbon electrode decorated by graphene–Prussian blue.

**Table 2:** Measured BHA concentrations in mayonnaise samples and recovery values.

Samples	Determined/mg kg^−1^	Recovery/%
1	19.02 ± 0.19	92–105
2	17.21 ± 0.12	95–102
3	17.94 ± 0.15	93–102

## Data Availability

Data are available from the authors on reasonable request.
